# Different Amounts of DNA in Newborn Cells of *Escherichia coli* Preclude a Role for the Chromosome in Size Control According to the “Adder” Model

**DOI:** 10.3389/fmicb.2018.00664

**Published:** 2018-04-05

**Authors:** Peter G. Huls, Norbert O. E. Vischer, Conrad L. Woldringh

**Affiliations:** ^1^Faculty of Science, Swammerdam Institute for Life Sciences, University of Amsterdam, Amsterdam, Netherlands; ^2^Bacterial Cell Biology, Swammerdam Institute for Life Sciences, University of Amsterdam, Amsterdam, Netherlands

**Keywords:** *Escherichia coli*, deeply-constricted cells, newborn cells, DAPI-stained nucleoid, DNA segregation, adder growth model, ObjectJ

## Abstract

According to the recently-revived adder model for cell size control, newborn cells of *Escherichia coli* will grow and divide after having added a constant size or length, *ΔL*, irrespective of their size at birth. Assuming exponential elongation, this implies that large newborns will divide earlier than small ones. The molecular basis for the constant size increment is still unknown. As DNA replication and cell growth are coordinated, the constant *ΔL* could be based on duplication of an equal amount of DNA, *ΔG*, present in newborn cells. To test this idea, we measured amounts of DNA and lengths of nucleoids in DAPI-stained cells growing in batch culture at slow and fast rates. Deeply-constricted cells were divided in two subpopulations of longer and shorter lengths than average; these were considered to represent large and small prospective daughter cells, respectively. While at slow growth, large and small prospective daughter cells contained similar amounts of DNA, fast growing cells with multiforked replicating chromosomes, showed a significantly higher amount of DNA (20%) in the larger cells. This observation precludes the hypothesis that Δ*L* is based on the synthesis of a constant *ΔG*. Growth curves were constructed for siblings generated by asymmetric division and growing according to the adder model. Under the assumption that all cells at the same growth rate exhibit the same time between initiation of DNA replication and cell division (i.e., constant *C+D*-period), the constructions predict that initiation occurs at different sizes (*Li*) and that, at fast growth, large newborn cells transiently contain more DNA than small newborns, in accordance with the observations. Because the state of segregation, measured as the distance between separated nucleoids, was found to be more advanced in larger deeply-constricted cells, we propose that in larger newborns nucleoid separation occurs faster and at a shorter length, allowing them to divide earlier. We propose a composite model in which both differential initiation and segregation leads to an adder-like behavior of large and small newborn cells.

## Introduction

The early ideas of Koppes et al. (1978a,b) and Voorn et al. (1993) that *Escherichia coli* cells grow by adding a constant length between divisions, were based on measurements of cell lengths and the rate of DNA replication in pulse-labeled cells grown in batch culture and prepared for electron microscopic autoradiography. This view has recently been revived in several studies (Amir, 2014; Campos et al., 2014; Jun and Taheri-Araghi, 2014); in the new experiments on cell size homeostasis (Campos et al., 2014; Taheri-Araghi et al., 2015; Wallden et al., 2016), the bacteria are grown in microfluidic chambers and observed by fluorescence light microscopy.

Whereas Koppes et al. (1978a,b) were able to measure only the length increment between initiation of DNA replication and the start of cell constriction, the extensive measurements of Jun and co-workers on large numbers of individual *E. coli* cells growing in a microfluidic “mother machine” (Wang et al., 2010) under a wide range of growth conditions covered the entire cell cycle (Taheri-Araghi et al., 2015). They confirm that at the population level, average cell size depends on growth rate exponentially (Schaechter et al., 1958); more importantly, they also show at the single cell level, that all cells in a particular growth medium grow in size at the same exponential rate and increase in size by the same amount (*ΔL*) between birth and division irrespective of their newborn size. Consequently, a large newborn cell will synthesize *ΔL* faster and will divide at a slightly earlier age than a small newborn cell, thus contributing to homeostasis (Figure 3 in Taheri-Araghi et al., 2015).

In several recent studies it has been discussed that the chromosome could play a role in establishing the constant size increment inherent to the adder model (Campos et al., 2014; Robert, 2015). Such constancy could be based on the chromosome serving as a “measuring stick” if newborn cells contain the same amount of DNA independent of their size at birth. For signaling cell division after duplicating this amount of DNA, a tight relation would have to exist between nucleoid replication/segregation and the peptidoglycan synthesizing machinery for cell division (Woldringh et al., 1991; Typas et al., 2012). This could be established via the so-called transertion process that involves transcription–translation and translocation of membrane proteins (Norris, 1995; Woldringh, 2002; Rabinovitch et al., 2003) and has been proposed to interfere with the assembly of the FtsZ-ring through nucleoid occlusion (Woldringh et al., 1991; Wu and Errington, 2012).

To detect whether newborn cells indeed contain equal amounts of DNA irrespective of their birth size, we have measured the DNA in nucleoids of large and small prospective daughter cells that can be assumed to give rise to large and small newborn cells. Cells were obtained from populations grown in batch cultures under steady state conditions at two different growth rates. As to be expected, only a small difference in DNA content (6%) was observed in newborn cells at slow growth. However, at fast growth and in the presence of multifork replication, large and small prospective daughter cells contained significantly different amounts of DNA (20%). This observation makes it unlikely that newborn cells base their constant length increment, *ΔL*, on the synthesis of equal amounts of DNA.

Graphical constructions of the adder cell cycle were made under the assumption that large and small newborn cells, generated by asymmetric division, have the same *C+D*-period. The constructions of length growth and genome content of single cells show that at fast growth large newborn cells exhibit a transient increase in the amount of DNA compared to small newborns, in agreement with the measurements. This lends support to the assumption that all individual newborns in a population have the same *C+D*-period and that large and small newborn cells initiate DNA replication at different sizes. Moreover, the advanced state of segregation, measured as the distance between separated nucleoids, found in the larger deeply-constricted cells, allows for differential segregation and earlier division in the larger cells, as required by the adder model and for obtaining homeostasis.

## Materials and Methods

### Cells and Growth Medium

*Escherichia coli* strain PJ4271 (strain MC1000 transformed with pBR322) was grown at 37°C in MOPS-buffered minimal medium (Neidhardt et al., 1974) with 100 mg/ml ampicillin according to Jensen et al. (1999), except that NaCl was added (about 27 ml of 2 M NaCl to 500 ml of MOPS-medium) to increase the osmolality to 300 mOsm. For slow growth the medium was supplemented with succinate (4 g per l), giving a doubling time *Td* of 122 min. For rapid growth glucose (5 g per l) and 20 amino acids (at millimolar concentrations according to Neidhardt et al., 1974) were added, giving a *Td* of 29 min. Exponentially growing cultures with constant OD450/cell (determined with a Coulter counter) were grown to OD450 of 0.1 to 0.2 and processed for microscopy (cf. Stuger et al., 2002).

### Fluorescence Microscopy and Image Analysis

DNA was labeled by addition of DAPI (4′,6-diamino-2-phenylindole dihydrochloride, Molecular Probes) at a final concentration of 0.05 mg/ml to cells fixed with OsO_4_ (0.1% w/v). After at least 15 min the cells were concentrated by centrifugation (1 min at 13000 rpm) and attached to microscopy slides coated with a thin layer of 1% agarose in culture medium. Pictures were taken with an Olympus BX60 fluorescence microscope equipped with a 100W mercury lamp and a Princeton RTE 1317-k-1 cooled CCD-camera. To limit photobleaching and DNA damage by ultraviolet light we focused the cells in phase contrast mode before photography. In this way all cells were exposed to the same (limited) amount of UV light.

DNA content per cell (expressed in chromosome equivalents) was measured after mixing the fixed *E. coli* PJ4271 cells with fixed *E. coli pbpA*(Ts) cells that contain one, or two fully replicated chromosomes when grown into stationary phase at the permissive temperature (30°C) for at least 48 h (Vischer et al., 1999) and after staining the mixture with DAPI. The *pbpA* mutant cells used for calibration could be distinguished from the PJ4271 cells because of their larger diameter and spherical shape. Cells were measured using the program “Coli-Inspector”^1^. This is a specialized software package developed for the analysis of shape and fluorescence related properties of bacterial cells. The program runs in combination with ImageJ with plug-in ObjectJ (see Figure 1 in Vischer et al., 2015). Amounts of DNA were calculated by assuming that the value of integrated fluorescence per cell of the left peak of the DNA distribution of *pbpA* cells equals 1 chromosome equivalent (Supplementary Figures S1A,B; cf. Huls et al., 1999).

To calculate integrated fluorescence (see Vischer et al., 1999), the modal value of the entire image was considered as background and was subtracted before subsequent image analysis.

### Determination of *C* and *C+D*-Periods

For the construction of the cell cycles at the two growth rates (succinate with Td = 122 min and glucose plus amino acids with Td = 29 min), the *C-* and *D*-periods have to be known. These were determined by image-cytometric measurement of the amount of DNA per cell during run-off DNA synthesis after inhibiting initiations with 300 μg/ml rifampicin, as previously described (see Figure 3 in Huls et al., 1999). In similar experiments the accumulation of DNA per cell (and per nucleoid) reached a plateau value after about 70 or 53 min in succinate or glucose plus amino acids medium, respectively. With these population values of the *C*-periods, the *D*-periods were subsequently calculated from the values of average chromosome equivalents per cell *G_c_*, using the expression G_c_ = {T_d_/C.ln2}{2^(C+D)/Td^-2^D/Td^} (Cooper and Helmstetter, 1968; Bremer and Churchward, 1977). It should be emphasized that this formula is only applicable to cell populations in steady-state growth. In our experiments this was verified by a constancy of average cell mass (OD450/cell counts) during the 5 h preceding cell sampling.

From the experimental values for *G_c_* of 1.5 and 3.7 chromosome equivalents per cell in the slowly (Td = 122 min) and rapidly (Td = 29 min) growing populations, the *D*-periods were calculated to be 40 and 25 min (cf. **Figures 2B,D** below). The values are comparable with those reported by Huls et al. (1999); note that the growth temperature in those experiments was 28°C, whereas it is 37°C in the present study. Variations in the values for *C-* and *D*-periods have also been reported for *E. coli* K-12 strains grown at 30°C (Michelsen et al., 2003).

## Results

### Measurement of DAPI-Stained Nucleoids in Prospective Daughter Cells

To determine the amount of DNA in large and small prospective daughter cells, we stained fixed cells with DAPI and measured the fluorescence of the nucleoids and the size of the cells in fast- and slow-growing populations. From the subpopulations of constricting cells we sampled the deeply-constricted cells, defined as those having a diameter at the constriction site that is smaller than the mean constriction diameter (**Table 1**, column 9). These deeply-constricted cells that were just about to divide, were assumed to represent prospective newborn cells (see Grover and Woldringh, 2001, for a similar analysis). Although the average length of the deeply-constricted cells with respect to all constricting cells had slightly increased (by 1–4%; see **Table 1**, compare columns 7 and 10), they largely covered the range of all constricting cells (Supplementary Figure S2), indicating a limited elongation during the constriction process.

**Table 1:** Properties of small and large newborn cells as estimated from deeply-constricted cells assumed to represent prospective daughter cells.

Growth medium (Td)	Total population			Constricted cells^(1)^					Deeply-constricted cells (diameter at constriction site < mean diameter at constriction site^(2)^)				
	Cell count	Mean length, μm	Mean diameter, μm	% Constricted cells (count)	Constriction period *T*, min	Mean cell length, μm (CV^3^)	Mean cell diameter, μm	Mean diameter at constriction site, μm	Mean length, μm (count)	Amount of DNA^(4)^ arbitrary units ± SD (count)		Nucleoid length^(5)^ μm ±*SD* (count)	
										Small^(6)^ (count)	Large^(6)^ (count)	Small^(6)^ (count)	Large^(6)^ (count)
**1**	**2**	**3**	**4**	**5**	**6**	**7**	**8**	**9**	**10**	**11**	**12**	**13**	**14**

Succinate (122 min)	1022	2.78	0.63	9 (95)	15	3.67 (10%)	0.6 (8%)	0.5	3.7 (51)	0.36 ± 0.03 (24)	0.38 ± 0.03 (27)	0.98 ± 0.09 (24)	1.06 ± 0.11 (26)
Glucose+aa (29 min)	772	3.24	0.88	26 (198)	10	4.12 (13%)	0.9 (6%)	0.7	4.3 (119)	0.62 ± 0.08 (63)	0.75 ± 0.12 (56)	1.19 ± 0.14 (62)	1.45 ± 0.19 (55)

*Parameters are shown of length distributions of constricted cells for two populations of *E. coli* MC4100, with values of fluorescence of DAPI-stained nucleoids and of nucleoid lengths. ^*(1)*^Automatically measured by the ImageJ plug-in ObjectJ using the program “Coli-Inspector.” The constriction period, *T*, was calculated from *T* = *Td*.ln(*1+F*)/ln2, in which *F* is the fraction of constricted cells (based on Bremer and Churchward, 1977). ^*(2)*^Constricting cells with a diameter at the constriction site smaller than the mean diameter at the constriction site are considered to be deeply-constricted cells and assumed to represent prospective daughter cells. ^*(3)*^CV, coefficient of variation. ^*(4)*^Amount of DNA, automatically measured by ObjectJ using “Coli-Inspector,” and expressed as fluorescence brightness in arbitrary units. See profile plots in **Figures 3B,D**. ^*(5)*^Measured manually with ObjectJ. CV of nucleoid lengths varied between 9 and 14%. ^*(6)*^Small and large cells are, respectively, smaller or larger than mean length of deeply-constricted cells.*

**FIGURE 1:**
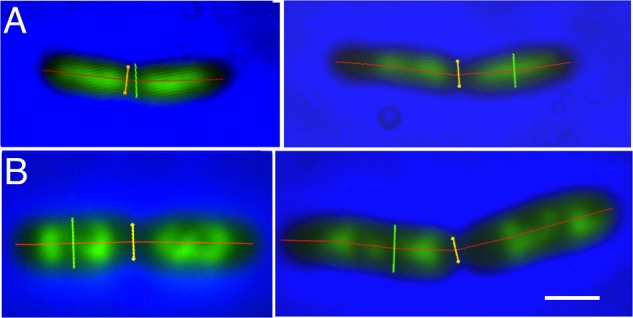
Examples of deeply-constricted *Escherichia coli* MC1000 cells, fixed with osmium tetroxide and stained with DAPI. Fluorescence images are overlaid with phase-contrast images and with markers, transiently displayed upon the images, for cell length (red), cell diameter (green) and the diameter at the constriction site (yellow), as measured by “Coli-Inspector” with ImageJ plugin ObjectJ. **(A)** Slow growing cells in succinate medium (Td = 122 min). Left and right panel, cell shorter and longer than 3.7 μm, respectively. **(B)** Fast growing cells in glucose plus amino acids medium (Td = 29 min). Left and right panel, cell shorter and longer than 4.3 μm, respectively. See mean lengths in **Table 1**, column 10. DAPI fluorescence is seen in green. Scale bar equals 1 μm.

**Figures 1A,B** show examples of deeply-constricted cells with their nucleoids from slow (Td = 122 min) and fast (Td = 29 min) growing cultures. The separated nucleoids can be seen to have a more extended shape in the longer constricted cells (right panels). As will be discussed below the distances between the segregated daughter nucleoids are larger in these cells.

**FIGURE 2:**
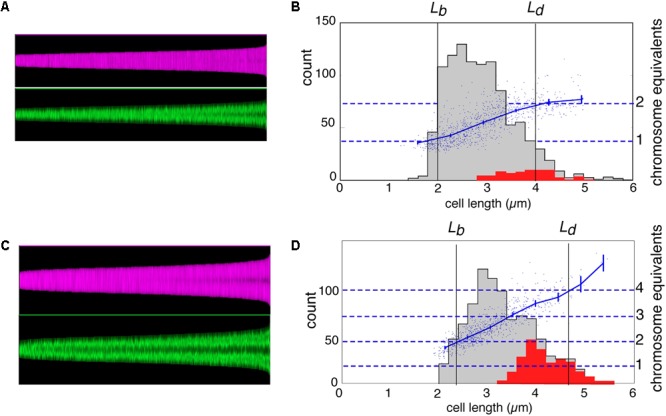
Maps of cell profiles and distributions of cell lengths. **(A,C)** Map of cell profiles, sorted according to ascending cell length. Each cell is visualized as a 1-pixel-wide column with a height corresponding to the cell length (in pixels) and showing local diameter (magenta) or the local fluorescence (green). The profile map visualizes the development of the constriction before cell division (magenta band becomes darker in the center due to the smaller diameter at the constriction site) and the change in fluorescence distribution of the (segregating) nucleoids along the cell length (green) (see Vischer et al., 2015). **(B,D)** Distribution of cell length of the total population (gray) and of constricting cells (red) with the lengths of newborn (*Lb*) and dividing cells (*Ld*) indicated. *Lb* was calculated from *Lb* = < *L > /2ln2*, in which < *L* > is the mean length of all cells, under the assumption of exponential elongation. In addition, the total DAPI-fluorescence per cell is shown by a scatter plot, with a line through the averages of binned data with vertical 95% confidence error bars in blue (in chromosome equivalents, right-hand ordinate). **(A,B)** Slow growing cells in succinate medium (Td = 122 min). **(C,D)** Fast growing cells in glucose plus amino acids medium (Td = 29 min).

In **Figures 2A,C**, the cell boundaries (magenta) and DNA fluorescence (green) of all individual cells in the two populations are arranged in so-called maps of cell profiles according to length, using the ImageJ plugin ObjectJ with software “Coli-Inspector” (see section “Materials and Methods”). In **Figures 2B,D**, histograms of all cells and of constricting cells (gray and red distributions, respectively) are shown together with a plot of the amount of DNA per cell (in chromosome equivalents) as a function of cell length. The plots indicate that in slow-growing cells (**Figure 2B**), DNA synthesis starts after a short *B*-period and slows down at the end, indicative of termination and a *D*-period. Because of overlapping DNA replication cycles (multifork replication) in the fast growing cells, such decreases in the rate of DNA synthesis are absent in **Figure 2D**.

**FIGURE 3:**
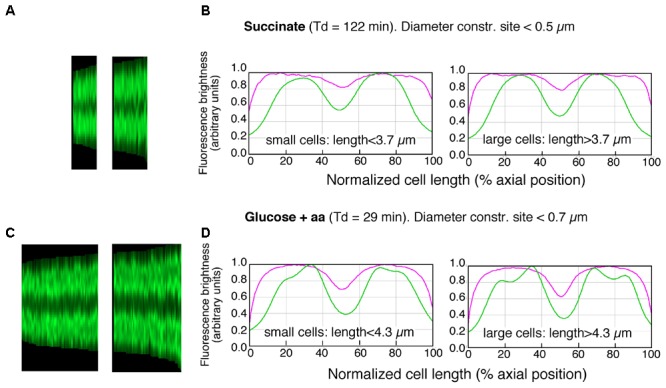
Maps of profiles and collective plots. **(A,C)** Maps of profiles of DAPI-fluorescence (cf. lower panels in **Figures 2A,C**) of deeply-constricted cells in slow **(A)** and fast **(C)** growing populations; subpopulations of small and large deeply-constricted cells are shown in left and right panels, respectively. **(B,D)** Collective profiles created from all cell profiles in the maps **(A,C)** by resampling to a normalized cell length of 100 data points and averaging to a single plot. Mean diameter- (magenta) and DAPI-fluorescence (green) are plotted in arbitrary units of brightness as a function of normalized cell length for slow **(B)** and fast **(D)** growing cells; subpopulations of small and large deeply-constricted cells are shown in left and right panels, respectively.

The maps of DNA profiles in **Figures 2A,C** illustrate the gradual elongation of both cells (magenta) and nucleoids (green). At fast growth a second segregation event can be seen to occur in the largest deeply-constricted cells (**Figures 3C,D**, right panels), indicative of re-initiation of DNA replication (see below).

To determine the amount of DNA in large and small newborn cells, we measured the integrated fluorescence per nucleoid and the cellular positions and lengths of the nucleoids for cells smaller and larger than the mean length of deeply-constricted cells, all of which contained two separated daughter nucleoids. The results are presented in **Figure 3** and **Table 1**. Because the nucleoids in the fast growing cells show rather irregular, lobular shapes characteristic of multifork replication (cf. **Figure 1B**), we also measured from the same cell populations nucleoid lengths manually (**Table 1**, columns 13 and 14).

Both the integrated fluorescence intensities, which represent the amount of DNA per nucleoid (**Figures 3B,D** and **Table 1**, columns 11 and 12), and the measurements of nucleoid lengths (**Table 1**, columns 13 and 14) indicate that the difference in DNA between small and large deeply-constricted cells at the fast growth rate is highly significant (*p* < 10^-8^, as determined by a two-tail *t*-test); at the slow growth rate, the difference remains significant, but just barely (*p* = 0.035). These differences are difficult to reconcile with a fixed amount of DNA (*ΔG*) serving as a constant in both large and small newborn cells, on which the constant length increment (*ΔL*) of the adder model could be based.

### The State of Nucleoid Segregation

The measurements of the amount of DNA in small and large constricting cells also give information on the state of segregation in deeply constricted cells, defined as the distance between their segregated nucleoids. The distances between nucleoids in deeply-constricted cells have been measured manually (**Table 2**, columns 4 and 5). Nucleoid separation was evaluated by eye for each individual cell. If some DAPI fluorescence could still be seen between nucleoids (usually after contrast enhancement) they were considered not to have segregated. In addition, distances between the centers of mass of segregated nucleoids have been calculated from the DNA profile plots as shown in **Figures 3B,D** (**Table 2**, columns 6 and 7).

**Table 2:** Distances between segregated nucleoids in deeply-constricted cells from slow and fast growing populations of *E. coli* MC1000.

Growth medium (Td min)	>Cells with two nucleoids		>Distances (μm) between nucleoids in deeply-constricted cells (diameter at constriction site < mean diameter at constriction site; see Table 1, columns 11 to 14)			
	% (cell count)	*S*-period^(1)^, min	>Measured manually^(2)^		>Distance between centers of mass measured from profile plots^(3)^	
			Small cells mean ±*SD* (cell count)	Large cells mean ±*SD* (cell count)	Small cells mean ±*SD* (cell count)	Large cells mean ±*SD* (cell count)
**1**	**2**	**3**	**4**	**5**	**6**	**7**

Succinate (122 min)	17 (1017)	24	0.56 ± 0.11 (24)	0.70 ± 0.14 (26)	1.26 ± 0.33 (25)	1.72 ± 0.21 (26)
Glucose+aa (29 min)	74 (747)	23	0.74 ± 0.12 (63)	0.89 ± 0.14 (56)	1.79 ± 0.15 (63)	2.21 ± 0.22 (56)

*^*(1)*^The period, *S*, during which cells contain two separated nucleoids, was calculated from *S* = *Td*.ln(1*+F*)/ln2, in which *F* is the fraction of cells with two nucleoids (based on Bremer and Churchward, 1977). ^*(2)*^Measured manually with ObjectJ program “Coli-Inspector.” ^*(3)*^For each cell the fluorescence profile along the cell axis (see **Figures 3A,C**) was subdivided in two parts left and right of the central minimum value (cf. **Figures 3B,D**). From either part, the center of mass was calculated from the region that exceeded the central minimum, and the distance between both was calculated.*

The results in **Table 2** (columns 4 and 5) indicate that the difference between the distances measured manually in the large and those in the small deeply-constricted cells at the fast growth rate, is very highly significant (*p* < 10^-8^). At the slow growth rate, the difference is still statistically significant but much less so (*p* = 0.0006). The same holds for the differences between the centers of mass (**Table 2**, columns 6 and 7) calculated from the profile plots for slow (*p* < 10^-6^) and fast growth (*p* < 10^-20^).

The present experimental set-up cannot provide information about the segregation-period of individual cells, *S* (the time during which two separated nucleoids exist or, in other words, the time between visible separation of daughter nucleoids and cell division). We can, however, determine the average *S-*period, on a population level, from the percentage of cells containing two nucleoids (**Table 2**, column 2, just as the duration of constriction, *T*, was calculated from the percentage of constricting cells (**Table 1**, column 6). Whether large and small newborn cells exhibit the same *S*- and *T*-periods can only be estimated from time-lapse studies (e.g., Wallden et al., 2016). In the Discussion we will argue that the more advanced state of segregation at the end of the cell cycle of large, deeply-constricted cells could result from the larger space available to the nucleoids when being pushed apart in the long cell axis by the invaginating envelope during the constriction process (Supplementary Figure S3).

### Generation of Large and Small Newborn Cells

Different newborn cell sizes can be assumed to originate either from stochastically postponed or premature symmetric divisions, or from asymmetric cell divisions. We do not know what their relative contributions are to the length distributions in the present populations. However, the coefficients of variation of the so-called *K(L)* distributions (length of prospective daughter cell/length constricting cell; Trueba, 1982), indicative of the degree of asymmetry, were found to be 9.5 and 4.9% for the succinate and glucose+amino acids populations, respectively. This suggests that asymmetric divisions do contribute to the subpopulations of large and small prospective daughter cells that we consider here.

**FIGURE 4:**
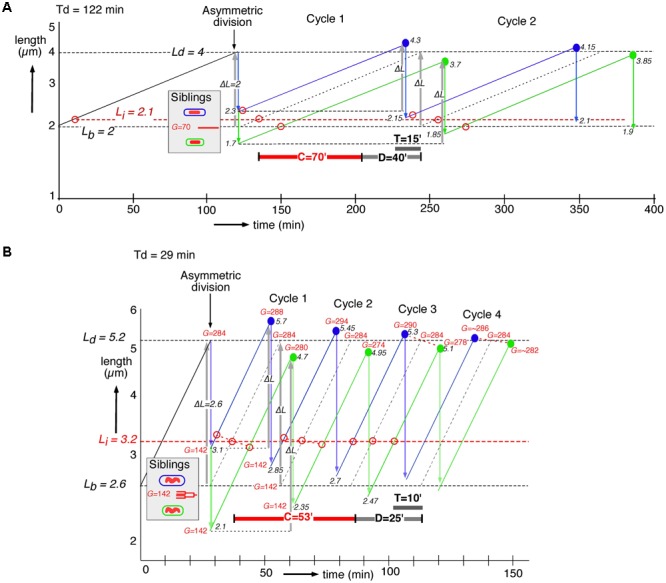
Construction of length growth curves of large (blue lines) and small (green lines) siblings, generated by asymmetric division. Cells elongate exponentially (elongation rate proportional to length); note log scale of the ordinate. Cell lengths can be considered to represent cell volumes if cell diameter is assumed to remain constant during the cell cycle. The graphs are inspired by, but different from Figure 6 in Koppes et al. (1978b), where the constant length increment was assumed to occur between initiation of DNA replication and initiation of constriction. Average lengths at birth (*Lb*) and division (*Ld*) are indicated by dashed horizontal black lines. The first division of an average cell is assumed to occur asymmetrically producing a large (*Lb + 1 SD*) and a small (*Lb – 1 SD*) newborn cell. Each newborn cell is constrained to add a fixed length, *ΔL* (gray upward arrows) before division. Lengths at division for large and small newborn cells are indicated by solid blue and green circles, respectively; their subsequent symmetrical divisions are indicated by blue and green downward arrows. Black numbers refer to cell lengths *L* (μm) at division or birth. The time of initiation of DNA replication (open red circles) is determined by subtracting the *C+D*-period from the time of division dictated by the adder model. The original length at initiation (*Li*) is indicated by a dashed horizontal red line. Red numbers refer to genome content, *G*, expressed in minutes; division by *C* = 70 or 53 min gives *G* in genome equivalents. **(A)** An individual cell from a slow growing succinate population (Td = 122 min) with a length at birth of *Lb* = 2 μm, equal to the average length of newborn cells (CV = 15%), dividing after a size increment of *ΔL* = 2 μm. **(B)** Similar construction of an individual cell growing in glucose plus amino acids medium (Td = 29 min) with a length at birth *Lb* = 2.6 (CV = 15%) and a size increment of *ΔL* = 2.6 μm.

Siblings generated by asymmetric division will have the same DNA content (*G*) present in a larger or smaller cell volume (see shaded insert in **Figures 4A,B**). To understand their behavior in subsequent cell cycles, we constructed for slow and fast growing cells their growth curves assuming exponential elongation. Consequently, after adding a constant length (gray upward arrows, *ΔL*, in **Figure 4**), the large newborns divide at a younger age than the small newborns, thus decreasing the difference in the size at division and leading to homeostasis (Taheri-Araghi et al., 2015). In the graphs of the cell cycles of single slow and fast growing newborn cells, we also depict the time of initiation of DNA replication by subtracting the *C+D*-period from the time of division under the assumption that all newborn cells have the same *C+D*-period. The graphs show that the size differences at birth induced by the asymmetric division, become less at division, reaching steady state after about four cycles (**Figure 4B**), as predicted by the adder model (cf. **Figure 3** in Taheri-Araghi et al., 2015). The adder principle and the constant *C+D*-period cause initiations to occur at different lengths (*Li*, open red circles in **Figure 4**). These differences also return to the steady state of initiation length *Li* after about four cycles.

In the slow growing population with *C+D* = 110 min (**Figure 4A**), both large and small newborn cells can be seen to exhibit a short *B*-period and a relatively long *D*-period (cf. **Figure 2B**). In the fast growing population with *C+D* = 78 min (**Figure 4B**), large newborn cells will initiate very early in their cycle. This means that some newborn cells could have already initiated prior to their birth (i.e., in the previous generation), in accordance with the observation of a second segregation event in **Figures 3C,D** (right panels) and with the theoretical predictions of Taheri-Araghi (2015).

The average values of *C* and *D* measured for the two populations (see section “Materials and Methods”) were used to calculate the genome contents (*G*) of the dividing cells indicated in **Figure 4B**. It can be seen that after asymmetric division siblings with identical chromosomes and the same multifork chromosome structure, acquire different genome contents at their subsequent divisions (during about three cycles). It follows that the adder model together with the assumed constant *C+D*-period predict a transient increase of the amount of DNA in the progeny of large newborn cells (see red *G*-numbers in **Figure 4**), in qualitative agreement with the present observations (**Table 1**). The same behavior is obtained when constructing the cycles of large and small newborn cells after stochastically premature or postponed divisions, for instance, due to variations in the *C+D*-period.

## Discussion

According to the adder model (Amir, 2014; Campos et al., 2014; Osella et al., 2014; Taheri-Araghi et al., 2015), individual cells sense neither time nor absolute size but “measure” a fixed increase in length between divisions. The differences in size at division predicted by the three models, sizer, timer, and adder are rather small (Figure 5 in Zaritsky and Woldringh, 2015), but the observed positive correlation between sizes at division and at birth falsifies the sizer model, while the timer model is falsified by the negative correlation between generation time and size at birth (Figure 1D in Taheri-Araghi et al., 2015).

The adder mechanism requires that an individual cell monitor a property that is equal in all newborn cells. A constant total number of proteins per cell in different newborns and growth conditions were proposed to trigger cell division after reaching a threshold in each generation (Taheri-Araghi et al., 2015; Sauls et al., 2016). Alternatively, the absolute constant amount of DNA per nucleoid could serve as the signal. If equal in all newborn cells, the duplicating nucleoid could function as a “molecular ruler” to monitor a constant size increment (Campos et al., 2014; Zaritsky, 2015; Zaritsky and Woldringh, 2015). The present results, however, have shown that large newborn cells in a fast growing population, contain significantly more DNA (20%; **Table 1**) than small newborns, precluding the possibility that the adder model is based on duplication of a fixed amount of DNA in all newborn cells.

We based our observations of the amount of DNA in prospective daughters on the evaluation of the degree of constriction in fixed cells visualized by phase contrast microscopy using the software package of ObjectJ (see section “Materials and Methods”; for an elaborate analysis of constricting *E. coli* cells see Reshes et al., 2008). Classification of the degree of constriction has been performed previously on *E. coli* cells prepared by agar filtration and visualized by electron microscopy (Koppes and Nanninga, 1980; Grover et al., 1987; Vardi and Grover, 1993; Grover and Woldringh, 2001). However, subtle shape changes like the degree of constriction and the variation in cell width during cell elongation (Trueba and Woldringh, 1980) can also be observed using light microscopy of living or fixed (Supplementary Figure S5) cells. Such studies could prove very informative with large populations growing in a microfluidics device (Taheri-Araghi et al., 2015; Wallden et al., 2016).

To understand better how differences in the amount of DNA in large and small newborn cells could arise, we constructed length growth curves according to the adder model and calculated the genome contents (*G*) of large and small siblings that initiate DNA replication *C+D* min before division (**Figure 4**). Large newborn cells initiate earlier and at a larger length (*Li*) than small newborns (red open circles), and at fast growth they also develop transiently a higher genome content during the first three cycles (*G* and red numbers in **Figure 4B**). This qualitative agreement with the measurements of DNA contents (**Table 1**) supports the assumption of an equal *C+D*-period in all individual cells, consistent with the observations of Wallden et al. (2016). A mathematical framework for these graphical constructions has been presented before (Amir, 2014; Ho and Amir, 2015; Taheri-Araghi, 2015; Taheri-Araghi et al., 2015). The implied differential initiation could be explained by assuming that the differently-sized siblings, although born with identical chromosomes, have a different balance between the amount of their DNA and cytoplasmic initiators such as DnaA or other regulators (Hansen et al., 1991; Katayama et al., 2010; Skarstad and Katayama, 2013). The chromosome in the sibling with a larger volume could consequently initiate earlier than that in the smaller sibling if the cell contained more initiators. The open circles at Cycle 1 in **Figure 4** indicate that large and small siblings initiate at different lengths, deviating from the average length for initiation as indicated by *Li* (dashed horizontal red line in **Figure 4**). Here, cell length represents cell volume if diameter remains constant during the cell cycle. It should be noted that to our knowledge the earlier division in the larger sibling has not directly been observed in, for instance, time-lapse studies with cells growing in microfluidic channels (Taheri-Araghi et al., 2015; Wallden et al., 2016).

The question remains as to what the dominant mechanism is behind the adder phenomenon of cell growth. Do large and small newborn cells monitor their size (*ΔL*) from birth on, dividing after a fixed size increment that is based on some constant property other than the amount of DNA (cf. “birth-centric” view; Amir, 2016)? Or do different newborns initiate DNA replication at different sizes and divide after a constant *C+D*-period, causing large newborns to have shorter and small newborns longer generation times as though growing according to the adder principle? This “initiation-centric” or “adder-per-origin” model has been described by Ho and Amir (2015) and Zheng et al. (2016). The model is based on sensing the activity of initiation proteins (cf. Hansen et al., 1991) rather than on the need for cells to “measure” a size increment that depends on the synthesis of many macromolecules. However, the model still requires an additional timing mechanism for triggering cell division at the end of the *C+D*-period. As previously suggested (Campos et al., 2014; Zaritsky and Woldringh, 2015), this could be established by the sequence of events occurring in elongating cells starting with replication and the concomitant segregation of chromosome arms (Wiggins et al., 2010; Youngren et al., 2014; Woldringh et al., 2015; Männik et al., 2016) during the *C*-period. After termination and relief of nucleoid occlusion (Woldringh et al., 1991; Wu and Errington, 2012; Cambridge et al., 2014), the *D*-period starts by assembling the FtsZ-ring (den Blaauwen et al., 1999; Aarsman et al., 2005), followed by divisome maturation (van der Ploeg et al., 2013; Vischer et al., 2015) and visible cell constriction (*T*-period; Reshes et al., 2008; Tsukanov et al., 2011).

These latter processes, occurring during the *D*-period, can all be expected to proceed in a growth-rate dependent way. But because at higher growth rate cell width will increase, the duration of the joint processes of FtsZ-ring assembly, divisome maturation and polar-cap peptidoglycan synthesis, may remain the same. The effect of a higher growth rate is thus compensated by the increased surface of the polar cap to be synthesized, causing a constant *D-*period. Such phenomenon is supported by the observation that the *T*-period is more or less constant for doubling times shorter than 60 min (Woldringh et al., 1977; Zaritsky et al., 2006). Under conditions of thymine limitation, when cell width increases without a compensating change in growth rate (Zaritsky and Pritchard, 1973), the *D*-period has been found to increase (Zaritsky et al., 1999).

At slow growth (**Figure 4A**), the earlier initiation in the larger sibling and the constant *C+D*-period enable it to divide earlier in the first cycle than the average newborn cell. At fast growth (**Figure 4B**), the earlier initiation in the larger sibling will trigger a division event also after *C+D* min, but occurring in the *third* cycle. How then can the large sibling already divide earlier than the average newborn cell in the *first* cycle, as dictated by the adder model and how does chromosome segregation accord with this adder principle? The unexpected observation of an increased distance (∼20%) between segregated nucleoids in large, deeply-constricted cells (**Table 2**) could explain the necessary acceleration of the cell cycle in large siblings. We envisage that a faster segregation of the nucleoid in the larger sibling could transiently occur because of the larger space along the length axis, accelerating the onset of constriction and division already in the first cycle. That the constriction process itself can enhance nucleoid separation has been demonstrated previously (Huls et al., 1999; Aarsman et al., 2005). It is also suggested here by the gradual increase in the distance between nucleoids during advancing constriction in both slow and fast growing cells (Supplementary Figure S3) and by the increased distance between separated nucleoids in constricting cells as a function of their length (Supplementary Figure S4). Whether the separation of daughter nucleoids also occurs faster in large elongating cells before the onset of constriction can only be ascertained by direct observation of live cells growing in microfluidics channels.

That cell volume regulates initiation of DNA replication seems well-established (Si et al., 2016; Zheng et al., 2016). That cell length regulates initiation of constriction has been proposed previously (Grover and Woldringh, 2001). This length model predicts the decrease observed in cell diameter during elongation (Trueba and Woldringh, 1980), and the correlations between cell dimensions and the coefficients of variation of cell length and volume at specific events like the onset of constriction. The proposal here thus comes down to a composite model in which both cell volume (for initiation of DNA replication) and cell length (for initiation of Z-ring assembly) play roles in determining cell division. Just as in the case of the larger sibling where the greater volume enables the chromosome to initiate earlier than average, its longer length enables faster segregation and earlier division, as dictated by the adder principle. Visualizing and measuring nucleoids at the single-cell level *in vivo* would establish whether or not differently-sized siblings indeed initiate DNA replication at different cell volumes and segregate nucleoids and initiate cell constrictions at different cell lengths. Such information could well be obtained using quantitative time-lapse imaging of mutants with enhanced asymmetric division (Männik et al., 2017).

## Author Contributions

PH performed the research and reviewed the manuscript. NV analyzed the data and reviewed the manuscript. CW wrote the paper.

## Conflict of Interest Statement

The authors declare that the research was conducted in the absence of any commercial or financial relationships that could be construed as a potential conflict of interest.

## Footnotes

1 https://sils.fnwi.uva.nl/bcb/objectj/examples/Coli-Inspector/Coli-Inspector-MD/coli-inspector.html
