# Supplementary material for: Different Amounts of DNA in Newborn Cells of Escherichia coli Preclude a Role for the Chromosome in Size Control According to the “Adder” Model
Source: Front Microbiol. 2018 Apr 5;9:664. doi: 10.3389/fmicb.2018.00664 (PMC5895768; doi:10.3389/fmicb.2018.00664)
Supplement: Supplementary file 1 [file Data_Sheet_1.PDF]

Supplementary Material

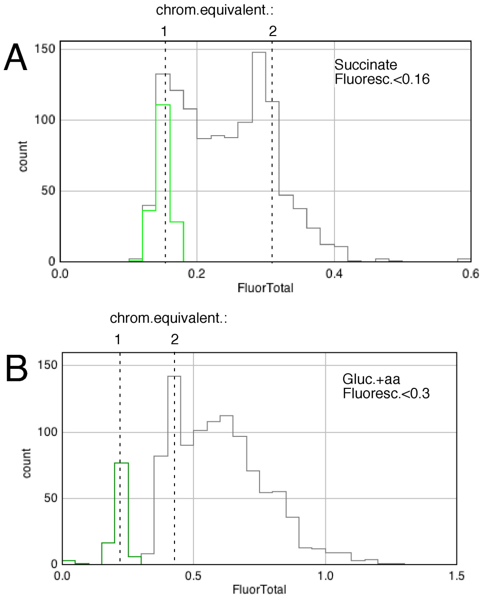

Figure S1. Distributions of total fluorescence per cell in the calibration cells (pbpA-mutant; green) and in the *E. coli* PJ4271 cells grown in **(A)** succinate and **(B)** glucose plus amino acids medium. The peak of the pbpA distribution is assumed to represent 1 chromosome equivalent as shown above the graphs. Note different scales for total fluorescence.

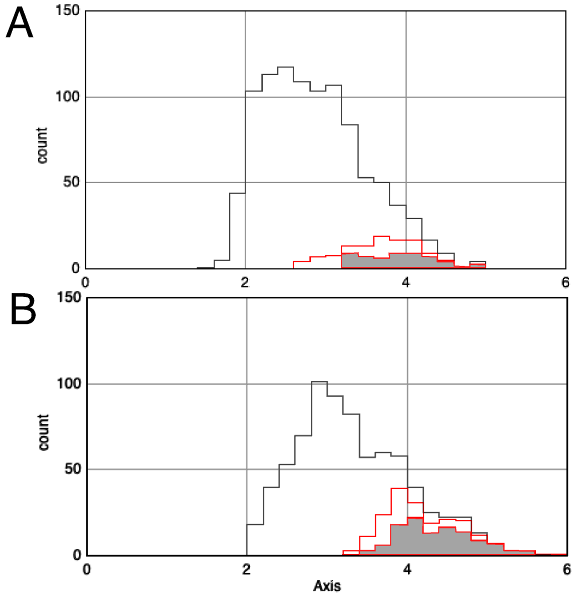

Figure S2. Length distributions of cells grown **(A)** in succinate and **(B)** in glucose plus amino acids medium. Distributions in red are from constricting cells, shaded distributions from the deeply-constricted cells (see Table 1), showing that they largely cover the range of all constricting cells.

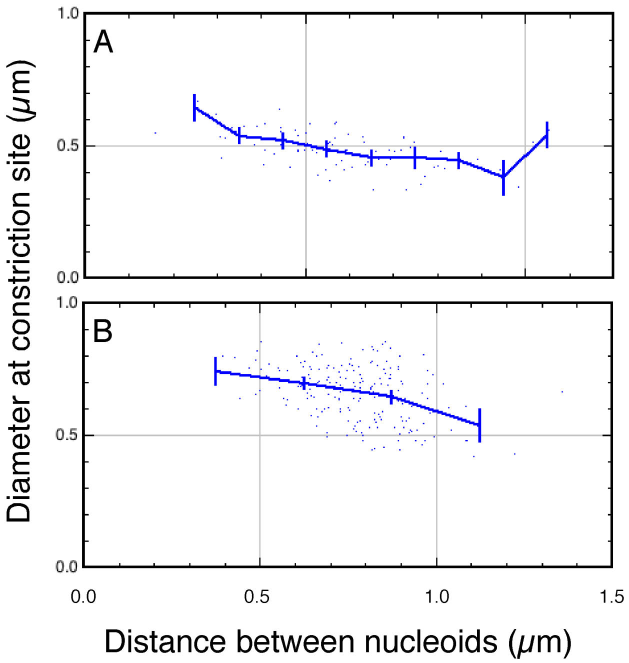

Figure S3. Scatter plots of degree of constriction versus distance between separated nucleoids in cells grown **(A)** in succinate and **(B)** in glucose plus amino acids medium with a line through the averages of binned data and with vertical 95% confidence error bars. The graph is interpreted as indicating that advancing constriction (smaller diameter at constriction site) causes a larger distance between daughter nucleoids.

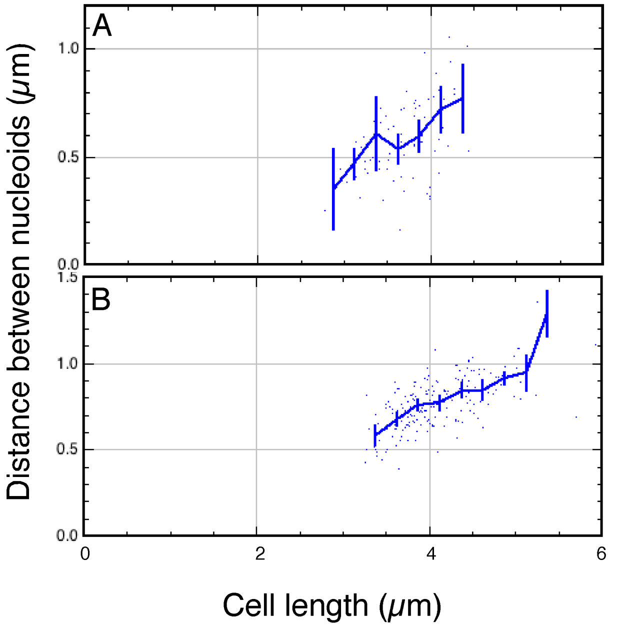

Figure S4. Scatter plots of distance between separated nucleoids as a function of cell length in constricting cells grown **(A)** in succinate and **(B)** in glucose plus amino acids medium with a line through the averages of binned data and with vertical 95% confidence error bars.

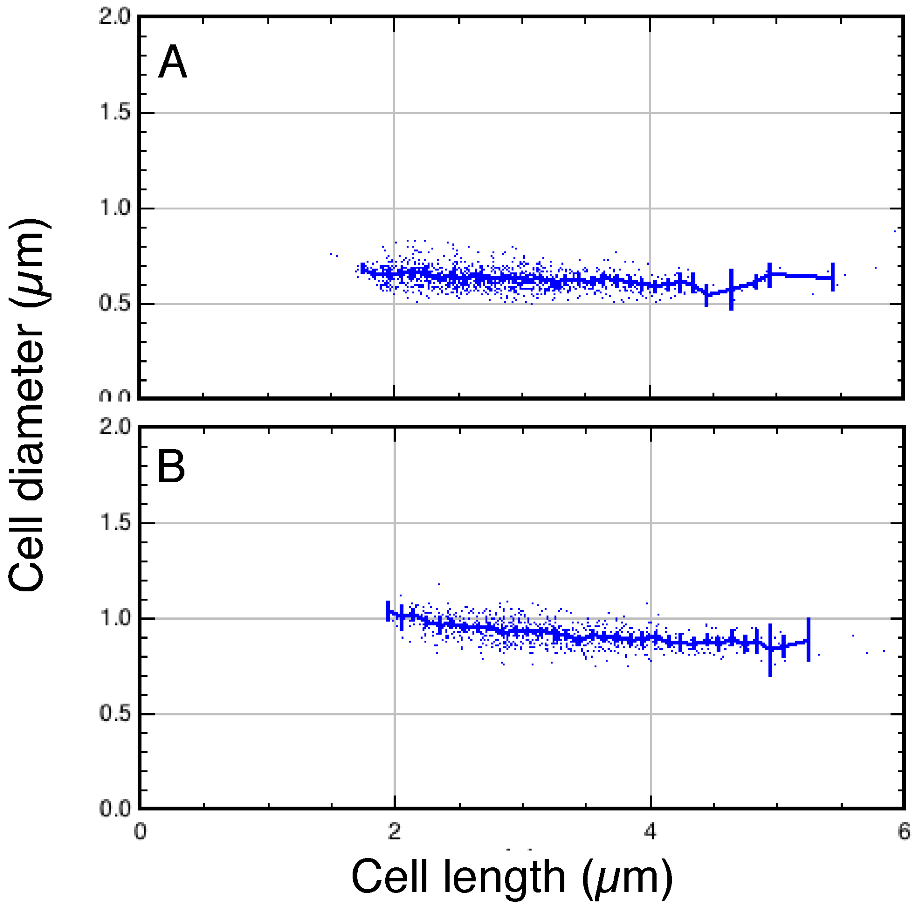

Figure S5. Diameter versus length plots of cells **grown (A)** in succinate and **(B)** in glucose plus amino acids medium with a line through the averages of binned data and with vertical 95% confidence error bars.
